# The Profile of Emotional Competence (PEC): A French short version for cancer patients

**DOI:** 10.1371/journal.pone.0232706

**Published:** 2020-06-18

**Authors:** Anne-Sophie Baudry, Veronique Christophe, Emilie Constant, Guillaume Piessen, Amelie Anota, the FREGAT Working Group

**Affiliations:** 1 Pôle cancérologie et spécialités médicales—Centre Hospitalier de Valenciennes, Valenciennes, France; 2 Univ. Lille, CNRS, CHU Lille, UMR 9193—SCALab—Sciences Cognitives et Sciences Affectives, Lille, France; 3 French National Platform Quality of Life and Cancer, France; 4 Human and Social Sciences Department, Centre Léon Bérard, Lyon, France; 5 Department of Digestive and Oncological Surgery, University of Lille, Claude Huriez University Hospital, Lille, France; 6 Methodology and Quality of Life in Oncology Unit (INSERM UMR 1098), University Hospital of Besançon, Besançon, France; Iranian Institute for Health Sciences Research, ISLAMIC REPUBLIC OF IRAN

## Abstract

**Background:**

Intrapersonal and interpersonal Emotional Competence (EC) predicts better health and disease adjustment. This study aimed to validate a short version of the Profile of Emotional Competence (PEC) scale for cancer patients.

**Methods:**

Five hundred and thirty-five patients with cancer completed a self-reported questionnaire assessing their intra- and interpersonal EC (PEC), their anxiety and depression symptoms (HADS), and their health-related quality of life (QLQ-C30). Confirmatory factor analyses and Item Response Theory models with the Partial Credit Model were performed to validate and reduce the scale.

**Findings:**

The Short-PEC (13 items), composed of 2 sub-scores of intra- (6 items) and interpersonal (7 items) EC, showed an improved factorial structure (Root Mean Square Error of Approximation (RMSEA) = 0.075 (90% confidence interval 0.066–0.085), comparative fit index = 0.915) with good psychometric properties.

**Discussion:**

Future studies should use the Short-PEC to explain and predict the adjustment of cancer patients. The short-PEC could be also used in clinical routine to assess the level of EC of patients and to adapt psychosocial intervention.

## Introduction

Emotional competence (EC) used in daily life has been highlighted in the literature based on trait emotional intelligence. EC involves inter-individual differences in the processing of emotional information (e.g. the tendency to identify and understand emotions, to regulate emotions) [1]. EC involves taking the potential benefits from emotions (e.g. information, danger) and regulating dysfunctional emotions. In this way, it allows a better adaptation to the environment, especially in job performance [2], couple relationships [3], subjective well-being [4], and health [5,6].

In fact, EC is linked to better mental and physical health in the general and clinical population [5,6] as well as to better cognitive and affective well-being [4]. EC leads to the better adjustment of patients with cancer [7–9] or diabetes [10,11] for instance. EC affects people's health or adaptation to illness via several processes such as better health behaviors, fewer anxiety and depression symptoms, and better social support [7,12,13]. However, women tend to report better EC than men [1] and EC could have different effects in a stressful situation such as when facing disease [14].

Especially in the cancer context, EC is related to fewer anxiety and depression symptoms [5,7–9], fewer unmet supportive care needs [7] as well as a stronger internal locus of control [15,16], better social support [8], better quality of life [17], and life satisfaction [14]. Therefore, it is important to consider EC as a personal resource in cancer adaptation, in clinical practice and research.

In particular, intrapersonal—about one's own emotions—and interpersonal—about others' emotions—EC seem to have a different effect: for example, intrapersonal EC may have a greater influence on health and patients’ adjustment than interpersonal EC [1,5,7]. It is therefore essential to differentiate between intra- and interpersonal emotional processes in predicting health and patients' adjustment. To the best of our knowledge, there is only one scale that enables intra- and interpersonal EC to be assessed separately: the Profile of Emotional Competence (PEC) [1]. This is composed of 50 items with a 5-point response (1 “Strongly disagree” to 5 “Strongly agree”) validated in the general and French-speaking population. The questionnaire shows good psychometric properties with a good internal consistency for the scores of intra- and interpersonal EC (α = .90 for the two scores). This scale is used to assess how a person thinks he/she will use his/her EC in daily life. For example, does the person think he/she can easily find the words to describe his/her feelings? Or regain calm after a difficult event? This can be particularly crucial in an emotionally charged context such as cancer.

It is therefore important to validate a short version of this scale in clinical and cancer populations to reduce the fatigability of patients, who tend to report a high vulnerability. In addition, a short version could facilitate its use in clinical routine and its inclusion in studies in which multiple questionnaires need to be completed. Indeed, some authors consider scales of fifty or more items to be sub-optimal, particularly for studies with multiple scales or repeated measures or targeting participants who are likely to become bored or disengaged [18]. Ideally, a compromise should be found in the length of time taken to complete (long tests generally have better psychometric properties with greater domain coverage) and the demands placed on participants (e.g. long or repeated completions). Thus, a shortened version of the PEC will have the advantage of minimizing missing data and also answer theoretical questions regarding intrapersonal vs. interpersonal EC.

Thus, the objective of this study was to validate a shorter version of the PEC for a specific clinical population facing cancer. The first aim was to reduce the Full-PEC (PEC-50) and to improve the scale structure of the shorter version compared to that of the Full-PEC. The secondary aim was to assess and compare the construct validity and convergent/divergent validity of the short and full versions.

## Methods

### Sample and procedure

Five hundred and thirty-five patients participated in this study and completed a questionnaire before treatment, about 6 weeks after the diagnosis of cancer (*M* = 40.42 days; *SD* = 26.41). Data were collected via the Clinico-Biological Database FREGAT (French EsoGastric Tumors, https://www.fregat-database.org/en) from 30 centers in France [19]). The study was conducted in accordance with the Declaration of Helsinki and with authorization from the “Comité de Protection des Personnes Nord Ouest IV” (Ethics Committee, project number: 13/67). All participants provided their written informed consent.

### Measures

Participants completed a self-reported questionnaire assessing their sociodemographic data (e.g. age, gender, education level) and questionnaires assessing their EC, anxiety and depression symptoms as well as their health-related quality of life (HRQoL).

EC was assessed using the Profile of Emotional Competence (PEC) scale validated in French [1]. The Full-PEC is composed of 50 items with a 5-point response (1 “Strongly disagree” to 5 “Strongly agree”). It provides 2 scores: an intrapersonal score (25 items, e.g. “I find it difficult to handle my emotions”, “When I am touched by something, I immediately know what I feel”) and an interpersonal score (25 items, e.g. “I am good at sensing what others are feeling”, “Other people tend to confide in me about personal issues”) of EC used in daily life, based on the average of the corresponding items. Scores are estimated based on the mean response to each item of the corresponding component, and range from 1 to 5. Higher scores indicate a higher use of EC in daily life perceived by individuals.

The anxiety and depression symptoms were assessed using the Hospital Anxiety and Depression Scale (HADS) [20] validated in French [21]. The HADS is composed of 14 items with a 4-point response and provides 2 scores: an anxiety score (7 items) and a depression score (7 items). Each score is calculated from the sum of the responses to each item and ranges from 0 to 21. Higher scores indicate stronger symptoms of anxiety or depression.

The HRQoL was assessed using the EORTC QLQ-C30 specific to cancer patients validated in the French language [22]. It is composed of 30 items evaluating 15 dimensions of HRQoL. The present study was based on 2 items through a 7-point response related to the patients’ perception of their global health and quality of life status (HRQoL). The global HRQoL score ranges from 0 to 100 with a higher score indicating a better global HRQoL.

### Statistical analyses

A descriptive of the baseline sociodemographic and clinical characteristics of the patients was carried out. Qualitative variables were described using numbers and percentages. Quantitative variables were described using means with standard deviations (SD).

#### Sample size

For the confirmatory factor analysis, sample size was based on recommendations by Wolf, et al., (2013) suggesting that 500 patients is adequate for this analysis [23]. For the Item Response Theory (IRT) model, no clear rule has been proposed for sample size. However, a sample of 500 patients was considered sufficient for the estimation of fit statistics.

For the correlation analysis, a sample of 404 patients will produce a two-sided 95% confidence interval with a width equal to 0.10 when the estimate of Pearson's product-moment correlation is 0.70. A sample of 320 patients produces a two-sided 95% confidence interval with a width equal to 0.20 when the estimate of Pearson's product-moment correlation is 0.30.

#### Factor analyses

A confirmatory factor analysis (CFA) was carried out on the full scale (PEC-50) to evaluate empirically the scale structure, according to the intra- and interpersonal components of the questionnaire. Goodness of fit was assessed using the Chi-squared goodness-of-fit statistic, the ratio of the Chi-squared on the degree of freedom (df), and fit indexes including the Root Mean Square Error of Approximation (RMSEA) with its 90% confidence interval (acceptable value < 0.08), the standardized root mean square residual (SRMR, acceptable value < 0.08), the Comparative Fit Index (CFI, acceptable value > 0.90), the Tucker-Lewis Index (TLI, acceptable value > 0.90) and the Goodness of Fit Index (GFI, acceptable value >0.90) [24]. This analysis was performed on the complete case analysis (i.e. including patients with all items completed).

The scale was reduced using Item Response Theory (IRT) models. This is a modern psychometric approach increasingly used for questionnaire validation and reduction [25]. We chose to apply the Partial Credit Model (PCM), which is suitable for an ordinal responses scale. This model estimates an item difficulty parameter for each item category of response. The more difficult the item is, the more likely the patient is to choose a lower response to this item. In this study, the PCM was applied by component (intra and inter components). The adjustment of the model to the data was explored with global and individual item-fit statistics. A Bonferroni adjustment was used for individual *p*-values (considering the number of items in the corresponding component). If an adjusted *p*-value (*p* < .005) was significant, the corresponding item was suspected of not fitting the model expectations. Residual statistics (standardized values) were also examined. An item with a fit residual outside the range +/- 2.5 was suspected of not fitting the model. A high positive residual indicated unexpected response patterns, whereas a high negative residual indicated some redundancy with other items. A graph representing person abilities in parallel with item difficulties with the addition of the item information function was also examined to determine whether the scale could assess the EC of the target population. The scale is considered well-adapted to the target population if the item distribution covers the whole range of person abilities. This graph is called a person-item map.

The internal consistency of the components was estimated using the Person Separation Index (PSI). As for Cronbach’s alpha, a PSI ≥ 0.70 is considered acceptable [26].

An iterative procedure was chosen: at each step, the most problematic item for the scale according to the fit residuals was deleted, after agreement between three experts. If several items presented an abnormal fit residual, the discussion between the experts also took into account the meaning and the formulation of the item. Once no more items were problematic and the global adjustment was correct, the procedure was stopped.

The structure of the Short-PEC was then checked using CFA reporting the same goodness-of-fit as for the full scale. A comparison was made with the Full-PEC reporting the ratio of the Chi-squared on the degree of freedom (df) and Akaike Information Criterion (lower is better for both criteria) [27]. An Exploratory Factor Analysis was also explored in case the deletion of items impacts the global scale structure.

#### Psychometric properties

Pearson’s correlations were used to examine the construct validity of the Short-PEC with the other scales. EC, especially intrapersonal EC, should be correlated negatively with the anxiety and depression symptoms and the impaired HRQoL of patients, as previously found with the Full-PEC [7,17].

Kruskal-Wallis non-parametric tests were performed to compare the scores for the Short-PEC and the Full-PEC and to identify the discriminant validity with gender: women would report better EC [1].

All analyses were done using SAS (Version 9.4, SAS Institute Inc, Cary, NC), R (version 3.4.0), STATA (version SE.13) and RUMM2030 software. All tests were two-sided and the statistical significance level was fixed at 5% (no adjustment for multiple testing) except for individual *p*-values in IRT models.

## Results

### Participants

The questionnaire was completed by 535 patients, aged from 26 to 89 years (*M* = 63.48; *SD* = 11.20), with esophageal cancer (n = 242, 45% of the sample), gastric cancer (n = 175, 33% of the sample), or esophagogastric junction cancer (n = 118, 22% of the sample). The majority were men (78%), retired (60%), living in a couple (64%), and with a lower level of education (55%). A detailed sample description is provided in Table 1.

**Table 1:** Sample characteristics (*N* = 535).

Variable	*N*	%
*Mean age (range)*	63.48 (11.20)	26–89
*Gender*		
Female	119	22
Male	416	78
*Living*		
In a couple	341	64
Alone	104	20
Other	71	13
Missing data	6	3
*Employment status*		
Employed—active	46	9
Inactive	156	29
Retired	320	60
Missing data	11	2
*Education level*		
No certificate	50	9
Secondary education diploma—below baccalaureate	248	46
Baccalaureate or equivalent diploma	72	14
First undergraduate cycle degree or equivalent diploma	34	7
Second university cycle degree, doctorate or equivalent diploma	62	12
Missing data	66	12
*Cancer site*		
Esophageal	242	45
Gastric	175	33
Esophagogastric junction	118	22

### Factor analyses

#### The full scale (PEC-50)

The CFA analysis performed on the full scale highlighted relatively low goodness-of-fit statistics with a Chi-squared of 5.048, a RMSEA of 0.087 (not < 0.08) [90% CI 0.085–0.089], a SRMR of 0.12 (not <0.08), a CFI of 0.482 (not > 0.90), a TLI of 0.460 (not > 0.90), and a GFI of 0.43 (not >0.90).

#### Intrapersonal component of the Short-PEC

**Fig 1:**
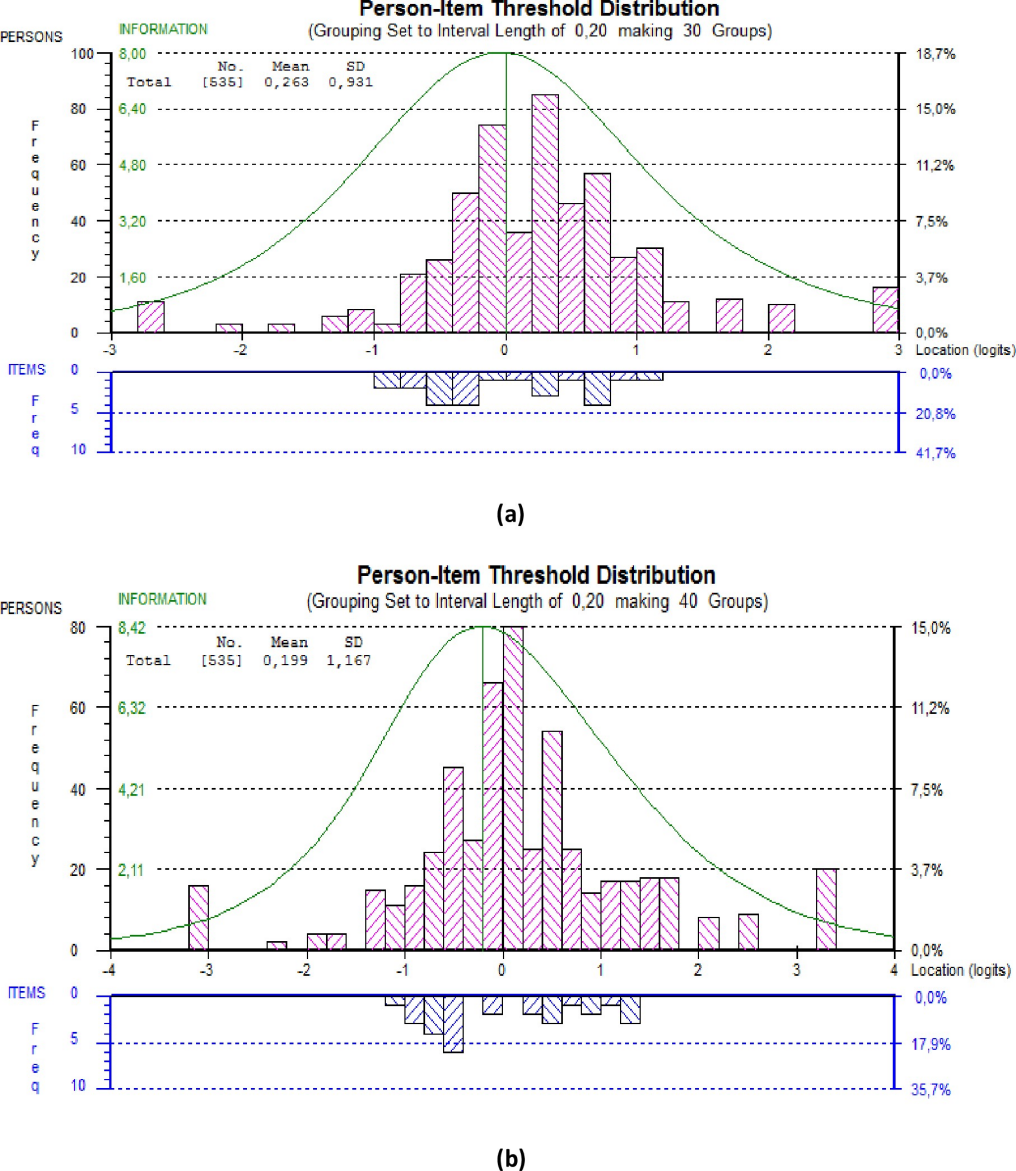
Person estimates and item estimates for the Short-PEC. a. Person estimates and item estimates for the Short-PEC intrapersonal component (6 items). b. Person estimates and item estimates for the Short-PEC interpersonal component (7 items). Person abilities represent the person level on the scale. The higher the person ability is, the higher the person’s score on the intrapersonal or interpersonal component of the Short-PEC. The parameters represented for items are difficulty parameters. Almost all the item difficulty parameters are between -1 and 1. The curve represents the total information curve. The scale is adapted to the population if the item distribution covers the whole range of person abilities.

Regarding the intrapersonal component of the Full-PEC, the IRT model highlighted poor goodness-of-fit statistics (global Chi-squared *p*-value < 0.001) with poorly fitting items. The PSI was equal to 0.756 (> 0.70). For example, item 9 (“I never base my personal life choices on my emotions”) was the most problematic with a fit residual equal to 9.640 > 2.5. This item was thus deleted after agreement between the experts. Then, the IRT model was rerun on the intrapersonal component scale without item 9. The same procedure was done for each problematic item in order to obtain finally a short intrapersonal component scale with no poorly fitting items and a good global goodness-of-fit statistic. Thus, the final short intrapersonal EC contained 6 items with an acceptable adjustment (global Chi-squared *p*-value = 0.021 < 0.05, Bonferroni adjusted *p*-value = 0.126) and a PSI of 0.749 (> 0.70), (Table 2). The person-item map (Fig 1A) highlights that the items are fully adapted to the population with the total item information curve covering the population.

**Table 2:** Item difficulties and fit statistics of the Short-PEC by component.

Items	Item difficulty	Standard error	Fit residual	*Adjusted p*-values
**Intrapersonal component**				
I am good at describing my feelings.	0.139	0.041	2.299	0.999
I easily manage to calm myself down after a difficult experience.	-0.091	0.043	-0.289	0.197
When I am sad, I find it easy to cheer myself up.	0.141	0.043	0.522	0.677
When I am touched by something, I immediately know what I feel.	-0.407	0.046	-0.018	0.999
If I dislike something, I manage to say so in a calm manner.	0.193	0.040	1.181	0.764
When I am angry, I find it easy to calm myself down.	0.026	0.041	1.593	0.409
**Interpersonal component**				
I can tell whether a person is angry, sad or happy even if they don't talk to me.	-0.376	0.045	1.949	0.999
I can easily explain the emotional responses of the people around me.	0.045	0.047	1.386	0.999
When I see someone who is stressed or anxious, I can easily calm them down.	0.234	0.047	0.239	0.999
Other people tend to confide in me about personal issues.	-0.113	0.043	0.351	0.999
I am good at sensing what others are feeling.	0.199	0.047	0.672	0.999
I am good at lifting other people's spirits.	0.072	0.047	-0.110	0.769
Other people tell me I make a good confidant.	-0.062	0.042	0.947	0.999

A Bonferroni adjustment was applied for *p*-values.

The more difficult the item is, the less likely patients are to choose a high positive response. An item with a fit residual outside the range +/- 2.5 was suspected of poorly fitting the model. A high positive residual indicated unexpected response patterns, whereas a high negative residual indicated some redundancy with other items.

#### Interpersonal component of the Short-PEC

Regarding the interpersonal component of the Full-PEC, the IRT model highlighted poor goodness-of-fit statistics (global Chi-squared *p*-value < 0.001) with poorly fitting items and a PSI of 0.799 (> 0.70). The same procedure was done as for the intrapersonal component scale. The final short interpersonal EC contained 7 items with an acceptable adjustment (global Chi-squared *p*-value = 0.230) and a PSI of 0.821 (> 0.70) (Table 2). These items are fully adapted to the population with the total item information curve covering the person abilities (Fig 1B).

#### The final Short-PEC (13 items)

**Fig 2:**
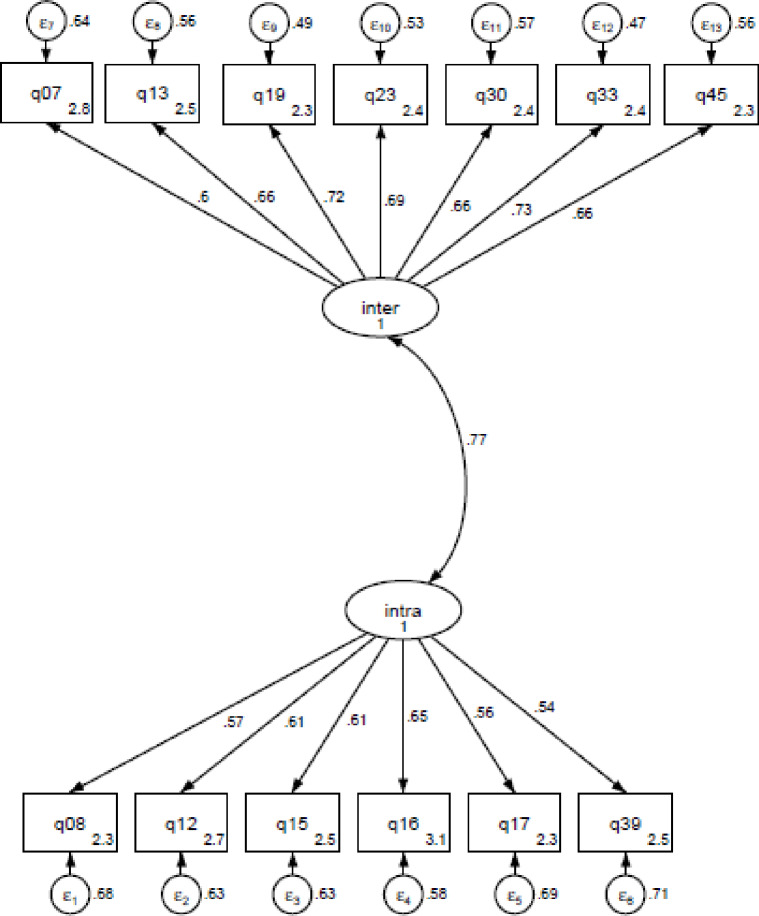
Confirmatory factor analysis of the Short-PEC. Adjustment of the model to the scale: Chi-squared = 4.033, RMSEA = 0.075 (< 0.08) [90% CI 0.066–0.085], a SRMR = 0.048 (<0.08), a TLI = 0.897 (< 0.90), a CFI = 0.915 (> 0.90), and a GFI = 0.89 (not >0.90).

Thus, the final Short-PEC contained 13 items (6 items of intrapersonal EC and 7 items of interpersonal EC) with a significantly improved factorial structure: a Chi-squared of 4.033, a Chi-squared/df of 4.03, an RMSEA of 0.075 (< 0.08) [90% CI 0.066–0.085], a SRMR of 0.048 (< 0.08), a TLI of 0.897 (< 0.90) and a CFI of 0.915 (> 0.90), and a GFI of 0.89 (not > 0.90) (Fig 2). For further details, the values of the deleted items have been added in a supplementary table (S1 Table). A comparison of the Full-PEC and Short-PEC support that the short-PEC has better structure than the Full scale (AIC = 21 408 for the Short-PEC vs. 86 191 for the Full-PEC, Chi-squared/df = 4.03 for the Short-PEC vs 5.05 for the Full-PEC). Since a lot of items have been deleted, an Exploratory Factor analysis was also performed in order to check if the global structure of the questionnaire has changed. This analysis revealed two components with same items correlated to the same components as for the CFA (data not shown).

#### Comparison between Full- and Short-PEC

The scores of intrapersonal EC did not differ for the Short-PEC (*M* = 3.31, *SD* = 0.88) and the Full-PEC (*M* = 3.27, *SD* = 0.49) (*p* = 0.217). However, the score of interpersonal EC for the Short-PEC (*M* = 3.21, *SD* = 0.96) was significantly higher than that for the Full-PEC (*M* = 3.03, *SD* = 0.51, *p* < 0.001).

### Psychometric properties

Overall, the results showed significant correlations between the two versions of the PEC (Short-PEC and Full-PEC) and the other scales: anxiety, depression, and HRQoL (Table 3). However, the Short-PEC seemed to be less associated with these variables than the Full-PEC.

**Table 3:** Correlations between age, anxiety, depression, HRQoL, and Short- and Full-PEC.

	Intra EC (Short-PEC)	Inter EC (Short-PEC)	Intra EC (Full-PEC)	Inter EC (Full-PEC)
Intra EC (Short-PEC)	1	0.615**	0.723**	0.464**
Inter EC (Short-PEC)	0.615**	1	0.558**	0.768**
Intra EC (Full-PEC)	0.723**	0.558**	1	0.622**
Inter EC (Full-PEC)	0.464**	0.768**	0.622**	1
Age	-0.045	-0.112**	-0.035	-0.141**
Anxiety	-0.158**	-0.000	-0.277**	-0.101*
Depression	-0.331**	-0.240**	-0.451**	-0.277**
HRQoL	0.169**	0.117**	0.231**	0.142**

* *p* < .05

**p < .01.

For the scores of intrapersonal EC, gender (women versus men) had no significant effect for both the Short-PEC (*M* = 3.30, *SD* = 0.86 and *M* = 3.31, *SD* = 0.88, respectively) and the Full-PEC (*M* = 3.30, *SD* = 0.50 and *M* = 3.27, *SD* = 0.40). For the scores of interpersonal EC, women reported higher scores than men for both the Short-PEC (*M* = 3.55, *SD* = 0.90 and *M* = 3.11, *SD* = 0.95, respectively, *p* < 0.001) and the Full-PEC (*M* = 3.17, *SD* = 0.51 and *M* = 2.99, *SD* = 0.50, respectively, *p* < 0.001).

## Discussion

The objective of this study was to validate a shorter version of the PEC that would be better adapted to the specific context of cancer patients. For this, we chose to use IRT models to reduce the Full-PEC. This is a modern psychometric approach, particularly suitable for scale construction and refinement, including the reduction of scales [28]. In fact, these models can highlight some redundant items or items not adapted to the full population targeted, which are the main criteria for item deletion. The obtained Short-PEC contains 13 items with a significantly improved factorial structure. This scale can assess the intrapersonal EC (6 items) and the interpersonal EC (7 items) used in daily life by cancer patients.

The previous structure of the Full-PEC (PEC-50) was confirmed with 2 components: intra- and interpersonal EC. However, the Full-PEC revealed relatively low goodness-of-fit statistics in the present sample of cancer patients. This finding could be explained by the population targeted. In fact, the Full-PEC was originally validated with students or young adults, reporting different EC scores from those of the general population [1]. In our study, the sample was composed of older people facing the disease. The Short-PEC also seems more adapted to cancer patients than the Full-PEC as highlighted by the person-item maps. This result confirms the importance of validating this Short-PEC scale on clinical populations, also to reduce the fatigability of patients and to facilitate its use in clinical routine and in studies.

The Short-PEC showed lower correlations with HRQoL, anxiety, and depression than the Full-PEC. This will therefore require external validation, which will be achieved by administering this short version to cancer patients (work in progress) and reassessing the correlation with anxiety/depression and HRQoL. It would probably also be interesting to study the psychometric properties of this short version in the general population.

Another important point for discussion is related to the fact that the interpersonal EC score for the Short-EC was significantly higher than that of the Full-EC. This could influence the comparison of the results of the studies using these two versions. However, because the IRT models provide a scale with items that are better adapted to the target population, it is possible that some items with lower scores in the Full-PEC may not have been well-adapted to cancer patients.

In clinical practice, the Short-PEC could be particularly important to get a baseline of patients' perception of their EC in daily life before starting psychosocial support. This can also quickly guide professionals to identify potential difficulties and to adapt interventions. The Short-PEC, which is easier to use, can replace the Full-PEC, particularly in routine clinical practice with repeated measurements. In addition, Short-PEC has been validated for cancer patients, unlike Full-PEC, which has been validated for general population.

## Limitations

This scale was validated in the specific context of patients with esogastric cancer, with a majority of men, and at the beginning of the cancer pathway. It may be necessary to verify the structure of the Short-PEC for other types of patients with cancer and at different steps of cancer care. In fact, gender, the context (e.g. a stressful situation or not, facing cancer) and the step of the cancer pathway have been shown to influence EC or its effect on disease adjustment [1,14,17].

## Conclusion

To conclude, the Short-PEC reveals good psychometric properties for clinical and research use. It enables the intra- and interpersonal EC used in daily life by cancer patients in a very emotional situation to be assessed. It is important to evaluate and reinforce their EC in psychosocial interventions to facilitate the cognitive and emotional processes required for a better adjustment to the disease and its treatments (e.g. emotional distress, quality of life, supportive care needs) [7,8,17].

## Supporting information

S1 File Full membership list of the FREGAT Working Group. (DOCX)

S2 File (DOCX)

S1 Table Values of the deleted items. (DOCX)
