# Supplementary material for: The Profile of Emotional Competence (PEC): A French short version for cancer patients
Source: PLoS One. 2020 Jun 18;15(6):e0232706. doi: 10.1371/journal.pone.0232706 (PMC7302700; doi:10.1371/journal.pone.0232706)
Supplement: S1 File — (DOCX) [file pone.0232706.s001.docx]

**The membership list of the FREGAT Working Group**

| **Center number** | **CENTERS** | **Family name** | **First name** |
| --- | --- | --- | --- |
| **1A** | Lille - CHU | PIESSEN | Guillaume |
| **1B** | Lille - Centre Oscar Lambret | EL HAJBI | Farid |
| **2** | Lyon  *(Pierre Bénite, Croix Rousse, Edouard Herriot)* | GLEHEN | Olivier |
| **3A** | Marseille - CHU Nord | D’JOURNO | Xavier Benoit |
| **3B** | Marseille - Institut Paoli Calmette | DELPERIO | Jean Robert |
| **4** | Villejuif | BENHAIM | Leonor |
| **5A** | Bordeaux - CHU - Chirurgie Digestive | JOUGON | Jacques |
| **5B** | Bordeaux – CHU - Chirurgie Viscérale | COLLET | Denis |
| **6A** | Toulouse – CHU Purpan | CARRERE | Nicolas |
| **6B** | Toulouse - Institut Claude Regaud | RIVES | Michel |
| **7** | Paris - HEGP | BERGER | Anne |
| **8** | Strasbourg – CHU Hautepierre | BRIGAND | Cécile |
| **9A** | Montpellier - CHU | FABRE | Jean Michel |
| **9B** | Montpellier - ICM | SAMALIN | Emmanuelle |
| **10** | Paris - St Antoine | PAYE | François |
| **11** | Bobigny - Avicennes | SABATE | Jean Marc |
| **12** | Clichy – CHU Beaujon | PANIS | Yves |
| **13** | Kremlin Bicêtre | PENNA | Christophe |
| **14** | Paris - Lariboisière | POCARD | Marc |
| **15** | Paris - Pitié Salpétrière | VAILLANT | Jean Christophe |
| **16** | Boulogne-Billancourt | PESCHAUD | Frédérique |
| **17A** | St Etienne - CHU Nord - Chirurgie Viscérale | PORCHERON | Jack |
| **17B** | St Etienne - CHU Nord - Chirurgie Thoracique | TIFFET | Olivier |
| **18A** | Nice - CLCC Lacassagne | FRANCOIS | Eric |
| **18B** | Nice - CHU Pasteur | MOUROUX | Jérome |
| **19A** | Clermont Ferrand - CHU Estaing | PEZET | Denis |
| **19B** | Clermont Ferrand – CLCC Jean Perrin | BENOIT | Cecine |
| **20** | Nîmes – CHU Caremeau | BORIE | Fréderic |
| **21A** | Nancy - CHU | GERMAIN | Adeline |
| **21B** | Nancy – Institut Cancérologie Lorraine | CONROY | Thierry |
| **22** | Reims - CHU | BOUCHE | Olivier |
| **23A** | Dijon - CHU | LEPAGE | Come |
| **23B** | Dijon - CLCC F.Leclerc | MAINGON | Philippe |
| **24** | Amiens - CHU Picardie | REGIMBEAU | Jean Marc |
| **25** | Limoges - CHU | MATHONNET | Muriel |
| **26** | Nantes - CHU | REGENET | Nicolas |
| **27A** | Caen - CLCC F. Baclesse | GALAIS | Marie Pierre |
| **27B** | Caen - CHU | LEBRETON | Gilles |
| **28** | Rouen - CHU C. Nicolle | DI FIORE | Fréderic |
| **29** | Grenoble - CHU Michaillon | BRICHON | Pierre-Yves |
| **30** | Tours - CHU | OUAISSI | Medhi |
| **31** | Colombes - Louis Mourier | MSIKA | Simon |
| **32** | Brest - CHU Morvan | METGES | Jean Philippe |
| **33A** | Rennes - CHU Pontchaillou | MEUNIER | Bernard |
| **33B** | Rennes - CLCC Eugène Marquis | PRACHT | Marc |
| **34** | Paris - Cochin | DOUSSET | Bertrand |
| **35** | Paris – Saint Louis | GORNET | Jean Marc |
| **36** | Besancon – CHU Jean Minjoz | PAQUETTE | Brice |
| **37** | Avignon – CLCC Sainte Catherine | MINEUR | Laurent |
| **38** | Nantes – ICO René Gauducheau | DUMONT | Frédéric |
