# Supplementary material for: The Profile of Emotional Competence (PEC): A French short version for cancer patients
Source: PLoS One. 2020 Jun 18;15(6):e0232706. doi: 10.1371/journal.pone.0232706 (PMC7302700; doi:10.1371/journal.pone.0232706)
Supplement: S2 File — (DOCX) [file pone.0232706.s002.docx]

Version courte du Profil des Compétences émotionnelles (S-PEC) des patients atteints de cancer

Les questions ci-dessous ont pour but de mieux comprendre comment vous vivez avec vos émotions. Répondez spontanément à chacune des questions en tenant compte de la manière dont vous réagissez en général ces dernières semaines. Il n'y a ni bonnes, ni mauvaises réponses car nous sommes tous différents à ce niveau. Pour chacune des phrases suivantes, entourez le chiffre qui correspond le plus à ce que vivez actuellement de 1 à 5. Le chiffre 1 signifie que la phrase écrite ne vous correspond pas du tout ou que vous ne réagissez jamais de cette façon. Le chiffre 5, au contraire, signifie que vous vous reconnaissez tout à fait dans ce qui est décrit ou que cela vous arrive très souvent.

Merci de bien répondre à toutes les questions dans l’ordre de présentation.

| *En général, ces dernières semaines…* | Pas du tout | | |  | |  | | Tout à fait | | |
| --- | --- | --- | --- | --- | --- | --- | --- | --- | --- | --- |
| 1. Je sais lorsqu’une personne est en colère, triste, ou joyeuse même si elle ne m’en parle pas. | | 1 | 2 | | 3 | | 4 | | 5 |  |
| 2. J’arrive facilement à trouver les mots pour décrire ce que je ressens. | | 1 | 2 | | 3 | | 4 | | 5 |  |
| 3.  Je retrouve facilement mon calme après avoir vécu un évènement difficile. | | 1 | 2 | | 3 | | 4 | | 5 |  |
| 4.  Je peux facilement expliquer les réactions émotionnelles de mes proches. | | 1 | 2 | | 3 | | 4 | | 5 |  |
| 5.  Quand je suis triste, il m’est facile de me remettre de bonne humeur. | | 1 | 2 | | 3 | | 4 | | 5 |  |
| 6.  Quand quelque chose me touche, je sais immédiatement ce que je ressens. | | 1 | 2 | | 3 | | 4 | | 5 |  |
| 7.  Si quelque chose me déplait, j’arrive à le dire calmement. | | 1 | 2 | | 3 | | 4 | | 5 |  |
| 8.  Quand je vois quelqu’un qui est stressé ou anxieux, il m’est facile de le calmer. | | 1 | 2 | | 3 | | 4 | | 5 |  |
| 9.  Les autres viennent facilement me parler de leurs problèmes personnels. | | 1 | 2 | | 3 | | 4 | | 5 |  |
| 10.  J’arrive facilement à savoir ce que les autres ressentent. | | 1 | 2 | | 3 | | 4 | | 5 |  |
| 11.  Je suis doué(e) pour remonter le moral des gens. | | 1 | 2 | | 3 | | 4 | | 5 |  |
| 12.  Quand je suis en colère, je peux facilement me calmer. | | 1 | 2 | | 3 | | 4 | | 5 |  |
| 13.  Les autres me disent que je suis un(e) bon(ne) confident(e). | | 1 | 2 | | 3 | | 4 | | 5 |  |

The Short Profile of Emotional Competence (S-PEC) for Cancer Patients

The questions below are designed to provide a better understanding of how you deal with your emotions in daily life. Please answer each question spontaneously, taking into account the way you would normally respond. There are no right or wrong answers as we are all different on this level.

For each question, you will have to give a score on a scale from 1 to 5, with 1 meaning that the statement does not describe you at all or you never respond like this, and 5 meaning that the statement describes you very well or that you experience this particular response very often.

| *In recent weeks …* |  | | |  | |  | |  | | |
| --- | --- | --- | --- | --- | --- | --- | --- | --- | --- | --- |
| 1. I can tell whether a person is angry, sad or happy even if they don't talk to me. | | 1 | 2 | | 3 | | 4 | | 5 |  |
| 2. I am good at describing my feelings. | | 1 | 2 | | 3 | | 4 | | 5 |  |
| 3.  I easily manage to calm myself down after a difficult experience. | | 1 | 2 | | 3 | | 4 | | 5 |  |
| 4. I can easily explain the emotional responses of the people around me. | | 1 | 2 | | 3 | | 4 | | 5 |  |
| 5.  When I am feeling low, I easily make a link between my feelings and a situation that affected me. | | 1 | 2 | | 3 | | 4 | | 5 |  |
| 6.  When I am touched by something, I immediately know what I feel. | | 1 | 2 | | 3 | | 4 | | 5 |  |
| 7.  If I dislike something, I manage to say so in a calm manner. | | 1 | 2 | | 3 | | 4 | | 5 |  |
| 8.  When I see someone who is stressed or anxious, I can easily calm them down. | | 1 | 2 | | 3 | | 4 | | 5 |  |
| 9.  Other people tend to confide in me about personal issues. | | 1 | 2 | | 3 | | 4 | | 5 |  |
| 10.  I am good at sensing what others are feeling.. | | 1 | 2 | | 3 | | 4 | | 5 |  |
| 11.  I am good at lifting other people's spirits. | | 1 | 2 | | 3 | | 4 | | 5 |  |
| 12.  When I am angry, I find it easy to calm myself down. | | 1 | 2 | | 3 | | 4 | | 5 |  |
| 13.  Other people tell me I make a good confidant. | | 1 | 2 | | 3 | | 4 | | 5 |  |
