# Supplementary material for: The Profile of Emotional Competence (PEC): A French short version for cancer patients
Source: PLoS One. 2020 Jun 18;15(6):e0232706. doi: 10.1371/journal.pone.0232706 (PMC7302700; doi:10.1371/journal.pone.0232706)
Supplement: S1 Table — (DOCX) [file pone.0232706.s003.docx]

**S1 Table. Values of the deleted items.**

|  |  |  |  |  |
| --- | --- | --- | --- | --- |
| **Item** | **Fit residual** | **df** | **Chi-squared** | ***P*-value** |
| **Intrapersonal EC component** |  |  |  |  |
| Item 9 | 9.64 | 509 | 202 | <0.001 |
| Item 10 | 6.52 | 509 | 36 | <0.001 |
| Item 20 | 5.86 | 508 | 55 | <0.001 |
| Item 21 | 9.57 | 507 | 187 | <0.001 |
| Item 22 | 13.83 | 506 | 306 | <0.001 |
| Item 48 | 5.64 | 504 | 43 | <0.001 |
| Item 24 | 9.79 | 503 | 157 | <0.001 |
| Item 41 | 6.13 | 501 | 50 | <0.001 |
| Item 6 | 4.276 | 499 | 41 | <0.001 |
| Item 38 | 4.04 | 497 | 16 | 0.048 |
| Item 49 | 4.5 | 494 | 14 | 0.095 |
| Item 25 | 3.99 | 491 | 35 | <0.001 |
| Item 42 | 4.13 | 487 | 18 | 0.021 |
| Item 26 | 3.68 | 484 | 28 | <0.001 |
| Item 43 | 4.84 | 479 | 21 | 0.008 |
| Item 37 | 4.38 | 472 | 21 | 0.008 |
| Item 2 | 3.56 | 465 | 41 | <0.001 |
| Item 1 | 7.55 | 456 | 124 | <0.001 |
|  |  |  |  |  |
| **Interpersonal EC component** |  |  |  |  |
| Item 3 | 2.89 | 510 | 75 | <0.001 |
| Item 36 | 2.58 | 510 | 43 | <0.001 |
| Item 4 | 3.9 | 507 | 60 | <0.001 |
| Item 11 | 3.83 | 507 | 48 | <0.001 |
| Item 5 | 6.07 | 504 | 28 | <0.001 |
| Item 18 | 5.87 | 504 | 56 | <0.001 |
| Item 27 | 5.15 | 501 | 36 | <0.001 |
| Item 40 | 5.83 | 499 | 92 | <0.001 |
| Item 46 | 6.89 | 498 | 92 | <0.001 |
| Item 44 | 7.69 | 496 | 122 | <0.001 |
| Item 28 | 8.42 | 493 | 99 | <0.001 |
| Item 34 | 9.78 | 490 | 216 | <0.001 |
| Item 31 | 12.14 | 487 | 283 | <0.001 |
| Item 29 | 14.94 | 483 | 367 | <0.001 |
| Item 47 | 3.4 | 464 | 8.66 | 0.37 |
| Item 32 | 3.77 | 456 | 8.06 | 0.43 |
